# Characterization and phylogenetic analysis of the mitochondrial genome sequence of *Heniochus acuminatus*

**DOI:** 10.1080/23802359.2022.2049016

**Published:** 2022-09-19

**Authors:** Fangyan Jiang, Ning Yang, Hai Huang

**Affiliations:** a Key Laboratory of Utilization and Conservation for Tropical Marine Bioresources of Ministry of Education, Hainan Tropical Ocean University, Sanya, China; b Key Laboratory of Tropical Marine Fishery Resources Protection and Utilization of Hainan Province, Hainan Tropical Ocean University, Sanya, China

**Keywords:** Mitochondrial genome, phylogenetic analysis, *Heniochus acuminatus*

## Abstract

In this study, the complete mitochondrial genome of *Heniochus acuminatus* was first sequenced and annotated. The entire mitogenome is 16,584 bp in length, which consists of 13 protein-coding genes (PCGs), 22 transfer RNA (tRNA) genes, two ribosomal RNA (rRNA) genes, and a non-coding control region. The phylogenetic analysis by maximum-likelihood (ML) method revealed that *H. acuminatus* belongs to the Chaetodontidae family and is closely related to other *Heniochus* fish. The complete mitochondrial genome of *H. acuminatus* is helpful in population genetics and molecular systematics.

*Heniochus acuminatus* (Linnaeus, 1758) is native to the Indo-Pacific region, where it is widespread and ranges broadly into tropical and subtropical regions (Kuiter 2002). It is an important aquarium fish distributed in coral reefs (Ohman et al. 1998). *H. acuminatus* is also one of the important bio-indicator species for assessing the health of coral reefs as they feed on coral polyps (Hourigan et al. 1988). To provide an efficient tool to study the biodiversity of this fish, we sequenced its mitochondrial genome.

The specimen of *H. acuminatus* was collected from Sanya Bay, Hainan, China (Lat 18°26′44″N; Long 109°50′03″E). COI (cytochrome c oxidase subunit I) gene sequence was used as a marker to identify the specimen as *H. acuminatus* (data not shown). Pectoral fins were stored in 75% ethanol at 4 °C. Total genomic DNA was extracted using the Animal Genome Extraction Kit (Invitrogen, Carlsbad, CA) following the manufacturers’ instructions. The specimen and DNA are stored in the museum of Hainan Tropical Ocean University (Dr. Huimin Feng, womenfeng@163.com) under voucher no. 2020H03. Our sampling procedure was approved by the Institutional Animal Care and Use Committee of Hainan Tropical Ocean University (approval ID 2020025). For Illumina pair-end sequencing of each sample, at least 1 μg genomic DNA was used for sequencing library construction. Paired-end libraries with insert sizes of ∼400 bp were prepared following Illumina’s standard genomic DNA library preparation procedure. The qualified Illumina pair-end library would be used for Illumina NovaSeq 6000 sequencing (150 bp × 2, Shanghai BIOZERON Co., Ltd., Shanghai, China). The mitochondrial genome was constructed using SPAdes v3.10.1 (Tolstoganov et al. 2019). The reads were assembled and annotated using MITOS (Bernt et al. 2013), and all protein-coding genes (PCGs) were aligned using the blastp. Finally, the phylogenetic analysis was performed using MEGA 6. A maximum-likelihood (ML) tree was constructed under the Kimura 2-parameter model with 1000 bootstrap replicates (Tamura et al. 2011).

Complete mitogenome sequences consisted of 16,584 bp for *H. acuminatus* (GenBank accession no. MW039154). It exhibits a slight A + T bias of 57.67%. It has a typical mitochondrial genome structure, consisting of 13 PCGs, 22 transfer RNA (tRNA) genes, two ribosomal RNA (rRNA) genes, and a non-coding control region. There are 16 intergenic spacer sequences totaling 156 bp (1–39 bp for each sequence) and four overlapping sequences totaling 22 bp (1–10 bp for each sequence), interspersed throughout the genome, and the largest (39 bp) spacer region is located between the tRNA^Asn^ and tRNA^Cys^ genes. Eight tRNA and ND6 genes are encoded on the L-strand, and the remaining genes are encoded on the heavy strand (H-strand). The length of all tRNAs ranges from 66 to 73 bp. There were four types of start codons and five types of stop codons. The four types of start codons were ATC (ATP8), ATA (ND5), GTG (COX1), and ATG of the other 10 genes. Five other types of stop codons are TAG (ND1, ND3), TGA (ATP8, COB), ND4 with incomplete codon T, and the remaining genes with the TAA codon. The 12S and 16S rRNA genes of *H. acuminatus* are 951 bp and 1645 bp, respectively.

The ML tree was constructed based on the complete mitogenome sequences from *H. acuminatus* and the other 13 closely related species in the GenBank database. According to Figure 1, we confirm that the *H. acuminatus* is much closer to *Heniochus diphreutes*, which coincides with the morphological taxonomy (Zuo and Tang 2011). The tree showed that *H. acuminatus* was grouped with other *Heniochus* fish in Chaetodontidae family and closely related to *Forcipiger flavissimus*.

**Figure 1.**
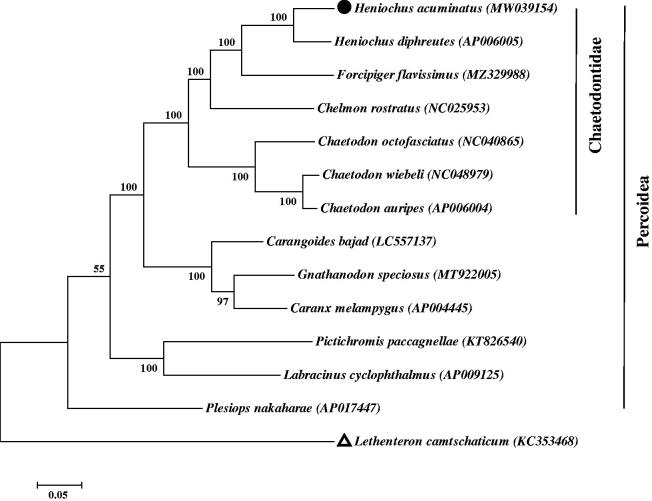
The maximum-likelihood (ML) tree of 13 Percoidea species is based on mitochondrial genomes with 1000 bootstrap replicates. The complete mitochondrial genome sequence was downloaded from GenBank. Accession numbers are indicated in parentheses after the scientific names of each species. The number at each node is the bootstrap value. The genome sequence in this study is labeled with a black spot, and *Lethenteron camtschaticum* as the outgroup is labeled with a triangle.

## Authors contributions

Fangyan Jiang was involved in the design, analysis of the data and the drafting of the paper. Yangning was involved in revising it critically for intellectual content. Hai Huang was involved in the final approval of the version to be published. The authors report no conflict of interest. The authors alone are responsible for the content and writing of the paper.

## Data Availability

The genome sequence data that support the findings of this study are openly available in GenBank of NCBI at https://www.ncbi.nlm.nih.gov/ under the accession no. MW039154. The associated BioProject, SRA, and Bio-Sample numbers are PRJNA735601, SRR14745269, and SAMN19589902, respectively.
